# Implementation of state health insurance benefit mandates for cancer-related fertility preservation: Following policy through a complex system

**DOI:** 10.21203/rs.3.rs-3340894/v1

**Published:** 2023-10-09

**Authors:** H. Irene Su, Bonnie Kaiser, Erika Crable, Ricardo Flores Ortega, Sara W Yoeun, Melina Economou, Estefania Fernandez, Sally AD Romero, Gregory A Aarons, Sara B. McMenamin

**Affiliations:** University of California San Diego Moores Cancer Center; University of California San Diego; University of California San Diego; University of California San Diego; University of California San Diego; University of California San Diego; University of California San Diego; University of California San Diego; University of California San Diego; University of California San Diego

## Abstract

**Background:**

A myriad of federal, state, and organizational policies are designed to improve access to evidence-based healthcare, but the impact of these policies likely varies due to contextual determinants, re-interpretations of and poor compliance with policy requirements throughout implementation. Strategies enhancing implementation and compliance with policy intent can improve population health. Critically assessing the multi-level environments where health policies and their related health services are implemented is essential to designing effective policy-level implementation strategies. California passed a 2019 health insurance benefit mandate requiring coverage of fertility preservation (FP) services for individuals at risk of infertility due to medical treatments to improve access to services that are otherwise cost-prohibitive. Our objective was to document and understand multi-level environment, relationships, and activities involved in using state benefit mandates to facilitate patient access to FP services.

**Methods:**

We conducted a mixed-methods study and used the policy-optimized Exploration, Preparation, Implementation, Sustainment (EPIS) framework to analyze implementation of California’s fertility preservation benefit mandate (SB 600) at and between the state insurance regulator, insurer and clinic levels.

**Results:**

Seventeen publicly available FP benefit mandate-relevant documents were reviewed, and four insurers, 25 financial, administrative and provider participants from 16 oncology and fertility clinics, three fertility pharmaceutical representatives, and two patient advocates were interviewed. The mandate and insurance regulator guidance represented two “Big P” (system level) policies that gave rise to a host of “little p” (organizational) policies by and between the regulator, insurers, clinics, and patients. Many little p policies were bridging factors to support implementation across levels and FP service access. Characterizing the mandate’s functions (i.e., policy goals) and forms (i.e., ways that policies were enacted) led to identification of (1) intended and unintended implementation, service, and patient outcomes; (2) implementation processes by level, EPIS phase; (3) actor-delineated key processes and heterogeneity among them; and (4) inner and outer context determinants that drove adaptations.

**Conclusions:**

Following the mid- and down-stream implementation of a state health insurance benefit mandate, data generated will enable development of policy level implementation strategies, evaluation of determinants and important outcomes of effective implementation, and design of future mandates to improve fit and fidelity.

## Introduction

Health policies can represent the evidence-informed implementation object, or be important facilitators of implementation of evidence-based practices (EBPs).^1^ Alternatively, policies that support health systems strengthening, for example via new infrastructure, workforce supports, or quality standards, have the potential to create the preconditions necessary to implement related EBPs.^2^ Research is needed to better understand how to capitalize on health systems-relevant policies that have the potential to support EBP access. Critically assessing the multi-level national, state, health systems, and organizational environments where health policies originate and wield influence is essential to understanding the roles that a policy can play in EBP implementation success and improving population health outcomes.^1^

The United States health care system is characterized by heterogeneous coverage of and access to specific health care services. To address this variation, the 2010 Affordable Care Act’s essential health benefits mandate began requiring all individual and small group health plans in the U.S. to provide insurance coverage for services described within ten categories deemed important to overall health and wellbeing (e.g., mental health, preventative care, maternal health). However, there has been nationwide variation in implementation of this mandate.^3–5^ In addition, the essential health benefits mandate does not apply to large group plans.^6^ This heterogeneity of covered benefits has led to the passage of subsequent federal and state benefit mandates that require health insurers to include specific health services in their benefit array. Benefit mandates are intended to regulate the health insurance market by standardizing benefit coverage of named services, thereby increasing access to specific health services at the population level and protecting consumers who might otherwise be vulnerable to under-provision of healthcare.^7^ Thus, benefit mandates can be conceptualized as policy innovations, or implementation objects aimed at promoting evidence-based care, reducing health disparities broadly, and advancing gender equality and reproductive rights specifically.^8^

Since 2017, many states have either passed (n = 16), are considering (n = 8), or have previously considered (n = 13) using benefit mandates to ensure access to fertility preservation (FP) services.^9^ FP benefit mandates facilitate access to evidence-based, standard-of-care treatments (e.g., oocyte, sperm, or embryo cryopreservation) for young people who are newly diagnosed with cancer and at risk of iatrogenic infertility (i.e., infertility that results from cancer treatment). Without benefit mandates, FP costs are high – averaging $10,078 for females to $468 for males – and typically not covered by health insurance.^10–13^ The flurry of legislative activity supporting FP benefit mandates highlights the need to specify the role these mandates play in implementation of widespread access to FP services.

The implementation of state FP benefit mandates occur across complex, multi-level health systems. In theory, once a state-level FP mandate is passed by the legislature and signed into law by the governor, insurance regulators should issue guidance on implementation. Health insurers should respond to the regulation by generating new benefit coverage, policies, and procedures to comply with the mandate. Oncology and fertility clinics should develop new contracts and protocols to interact with insurers and patients. Patients should then be able to access FP services with limited financial risk due to their new health insurance coverage. In reality, implementation of FP benefit mandates is no different than implementation of EBPs: implementation efforts are typically non-linear and characterized by fits and starts while individuals tasked with benefit implementation attempt to identify and mitigate barriers and ensure compliance. For example, in many states, health insurers with plans subject to FP benefit mandates had to comply with state legislation prior to regulator guidance being issued.^14^ Additionally, due to the U.S.’s federalist system, state legislation determining which specific services and populations are included under the purview of a FP benefit mandate varies across states, suggesting geographic variation and inequitable access to FP services.^14,15^ Implementation of a given FP benefit mandate requires multiple actors from state government agencies, health plans, and healthcare delivery organizations who can interpret and re-interpret what successful policy implementation means, thereby promoting additional variation in compliance and population health outcomes.^16^ How implementation occurs at each of these levels, and across outer systems contexts (e.g., legislature, insurance regulator) and inner organizational contexts (insurers and healthcare delivery organizations) can inform the types of multi-level implementation strategies that can support access to EBP FP services.

Among 16 states with FP benefit mandates, California’s mandate and insurance regulator guidance are among the least specific, with no details on what compliant coverage looks like.^14^ This allows heterogeneity in downstream implementation and highlights the need to understand how the mandate influences the multi-level health system and access to care for the more than 16 million individuals whose state-regulated health insurance is subject to the mandate.^13^ This lack of alignment across outer and inner context creates barriers to EBP implementation.^17^ To date, assessment of the multi-level, cross-context processes and outcomes of the FP benefit mandate has not been reported. Our objective was to use the policy-optimized Exploration, Preparation, Implementation, Sustainment (EPIS) framework to investigate California’s FP benefit mandate implementation in a health system comprised of state insurance regulator, insurers, and clinics and identify key policies, processes, and actors within and between contexts that impact access to mandated FP health insurance benefits. In doing so, we aimed to specify the features of a FP benefit mandate as a policy innovation and describe the policy’s fit and implementation in a complex multilevel state health system.

## Methods

Our team of implementation science, health policy, anthropology and clinical experts conducted a statewide mixed-methods study to assess processes and context factors in FP benefit mandate implementation among insurers, oncology and fertility clinics, and newly diagnosed cancer patients following the 2019 passage of California’s FP benefit mandate known as Senate Bill (SB) 600.^18^ Under the Knox-Keene Health Care Service Plan Act of 1975, health care service plans regulated by California’s Department of Managed Health Care are required to provide enrollees with basic health care services. SB600 states, “when a covered treatment may cause iatrogenic infertility to an enrollee, standard fertility preservation services are a basic healthcare service and are not within the scope of coverage for infertility treatment;” “provisions do not apply to Medi-Cal” (i.e., California Medicaid) contracts.^19^ The study was reviewed and approved by the Institutional Review Board at UC San Diego.

### Policy-optimized EPIS Framework

We applied recommendations for optimizing implementation science theories, models, and frameworks to tailor the EPIS framework to our study contexts and policy-relevant factors.^1^ EPIS was selected because it posits that implementation processes unfold across multiple levels through four non-linear phases.^20^ In the EPIS framework, determinants (i.e., implementation barriers and facilitators) across outer system and inner organizational contexts, and innovation factor characteristics (e.g., benefit mandate design) influence implementation activities and outcomes across these phases. Bridging factors spanning the outer and inner contexts facilitate alignment and implementation success across levels, including policy transfer. Crable et al.’s six recommendations for investigating policy and policy-level factors were used to augment our EPIS framework application to characterize the multi-level context and role FP benefit mandates play in FP service access.^1^ We specified where policy, systems, and service entities existed in multi-level contexts a priori, but used the recommendations to characterize the contexts and interrelationships among entities across contexts in implementation of the FP benefit mandate. Recommendations required the research team to: 1) specify a policy’s function; 2) specify a policy’s form(s); 3) identify and define nonlinear phases of implementation across outer and inner context; 4) describe the temporal roles that stakeholders play; 5) consider policy-relevant outer and inner context adaptations; and 6) identify and describe bridging factors – which can occur across outer-inner contexts and across different levels within the outer or inner context.

### Data Collection

We conducted document reviews of publicly available material pertaining to the process of developing, passing, and implementing the FP benefit mandate in California. This included a review of the following resources: reports to the California legislature prepared by the California Health Benefits Review Program; legislative text as posted on the California legislature’s legislative information (leginfo) website (leginfo.legislature.ca.gov); bill analysis as posted on the leginfo website (i.e., Assembly health bill analysis, Assembly appropriations bill analysis, etc.); and governor’s veto messages.

Between 2020 and 2022, we conducted semi-structured interviews about experiences with SB 600 and FP health insurance benefits with the following stakeholders: insurers; oncology and fertility clinical providers, social workers, and financial counselors from academic and community adult and pediatric oncology and female and male fertility programs; and cancer survivors who underwent FP in California. Due to ongoing litigation surrounding SB 600, the state regulator could not be interviewed; the study team instead reviewed publicly available documents.

Health insurers were selected from the Department of Managed Health Care’s full-service health plan’s enrollment report.^21^ Health insurers with more than 20,000 commercial enrollees were considered for inclusion in the study. FP services are utilized by < 0.1% of the population each year;^13^ thus, smaller plans may not have much experience with administering these benefits.

Clinical recruitment began with pediatric and adult oncologists and female and male fertility specialists at institutions affiliated with the UC San Diego or the Southern California Pediatric and Adolescent Cancer Survivorship Consortium, followed by snowball sampling of relevant stakeholders referred by these providers. Patient participants were recruited from consecutive cancer patients who underwent FP at one fertility clinic.

We conducted semi-structured video call interviews using interview guides based on two implementation science frameworks, EPIS and Bullock’s Policy Implementation framework.^1,20,22^ Interviews were audio-recorded and transcribed using Otter.ai software. Recruitment stopped when data saturation was achieved (i.e., additional interviews yielded no new insights).^23^

### Data analysis

We analyzed qualitative data in MaxQDA software (VERBI GmbH) using thematic analysis. We identified inductive themes, or those arising from the data, by having six researchers (BNK, EF, SY, SM, HS, RFO, ME) read the transcripts to become familiar with the text and develop initial codes. These were complemented by deductive themes drawn from Bullock’s model, which explicitly describes the process of shaping policy as it moves through multiple levels (i.e., state insurance regulator, insurer, and clinic), and the policy-optimized EPIS framework and in order to identify processes and related barriers and facilitators by level.^1,22^ Within each level, we sought to characterize implementation processes by EPIS phases: 1) Exploration, identification of the issue that requires policy-level intervention; 2) Preparation, development of policy package and subsequently interventions required to address the issue; 3) Implementation, the process through which intervention is implemented; and 4) Sustainment, continued use of intervention and inclusion into common practice. Next, three researchers (SY, RFO, ME) independently coded five transcripts iteratively and discussed disagreements, and the six-researcher team discussed ways to refine the codebook. Codes were applied to all transcripts using consensus coding (three coders independently coded each transcript and resolved discrepancies by consensus), maintaining rigor and reliability throughout the coding process. Code summaries were developed that synthesized each code.

## Results

In total, 17 documents were reviewed, and four insurers, 16 oncology and fertility clinics (contributing 25 participants: two clinical administrators, six financial counselors, 13 clinicians, and four patient navigators), three fertility pharmaceutical representatives, and two patient advocates participated. Findings are organized according to Crable’s six policy-relevant implementation science recommendations.^1^

### 1. Specify dimensions of a policy’s function: goals, policy type, contexts, resources/capital exchanged.

#### Policy Goal

As stated by bill author Senator Portantino, the goal of the benefit mandate was to improve access to FP services for patients undergoing cancer treatment.^24^ Specifically, the policy aimed at reducing denials of coverage for FP services and any related delays in providing cancer treatment to these patients by clarifying that FP services are basic health care services, and thus required to be covered by all DMHC-regulated health insurance products. Excluding insured individuals not subject to the mandate (Medi-Cal and self-insured) and uninsured individuals, the mandate applies to 42% of California’s overall population.^13^

#### Policy Type and Context

**Figure 1:**
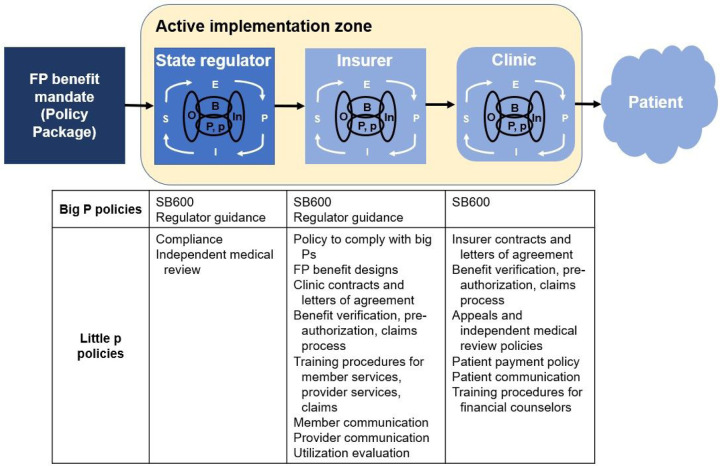
The multi-level active implementation zone for the fertility preservation (FP) benefit mandate. The implementation zone includes the state insurance regulator, insurers and clinics. Downstream of the Big P FP benefit mandate policy, both Big P and little p policies arise in regulator, insurer, and clinic implementation to enable patient access to these insurance benefits. Through multi-level implementation, these downstream policies reshape and dilute the policy package. *P = Big P, p = little p, E = Exploration, P = Preparation, I = Implementation, S = Sustainment, O = Outer context, In = Inner context, B = Bridging*

Treating the mandate as the EPIS innovation, its implementation occurs across three levels to ultimately reach patients who need to access these insurance benefits: regulator, health insurers, and clinics (Fig. 1); each level needs to implement policies related to the FP benefit mandate. The FP benefit mandate represents a “big P” policy to insurance regulators because it arises from a legislative body and requires compliance. Regulators then are responsible for issuing another “big P” policy when they issue guidance to the insurers for how to implement the mandate. Insurers need to comply with both the legislation and regulator guidance of “big P” or macro level policies. Downstream, in response to “big P” implementation, “little p” policies arise from regulators, insurers, and clinics and are implemented in level-specific contexts (Fig. 1). For example, in response to SB600 and the related regulator guidance, insurers can choose to be compliant by implementing a coverage policy to remove FP services from the list of excluded benefits in the evidence of coverage (EOC) documents. In response, clinics then generate new policies on additional benefit verification, pre-authorization, claims, and appeal efforts to seek coverage for patients.

#### Resources or Capital Exchanged

After SB 600 was signed into law, the regulator (DMHC) issued regulation as part of APL-20–001 on January 15, 2020 to detail compliance and filing requirements. The regulation defined applicable populations, affirmed coverage of ‘standard fertility preservation services’, and required that insurers submit documentation stating that all of their documents (current EOC, Summary of Benefits, Schedules of Benefits, Infertility Riders, Subscriber Agreements, and disclosure forms) did not specifically exclude FP benefits. If an insurer’s pre-mandate coverage policies were not in compliance as described above, the regulation required insurers to submit plans detailing future amendments to plan documents that would ensure timely compliance with SB 600.^25^ No resources were specifically allocated to the regulator or insurers for policy implementation. No financial support to comply with SB 600 was exchanged from state regulators to insurers. However, the policy created an opportunity for health insurers to reimburse contracted medical providers for the delivery of FP services to eligible members.

### 2. Specify dimensions of a policy's form: origin and creators, structural components, dynamism

#### Innovation Developers

In February 2011, California State Assembly member Portantino introduced the first known legislation, Assembly Bill (AB) 428, to require California health insurers to cover FP services.^13^ This policy innovation was further developed with information submitted by regulators, insurer groups, clinical groups, and patient advocacy groups. This bill was also supported by the American Society of Reproductive Medicine, California Medical Association, California National Organization for Women, Fertile Action, Medical Oncology Association of Southern California, and RESOLVE: The National Infertility Association. AB 428 died in the Assembly and was re-introduced in California three more times as AB 912 (2013), SB 172 (2017), and ultimately as SB 600 (2019). California eventually passed SB 600 as a FP mandate with a Democratic legislature and governor similar to the political environment in the other first states to pass FP mandates. More recent passage of FP mandates has occurred in states with Republican controlled legislature and/or governor.^9^

#### Innovation Characteristics

The earlier versions of the FP benefit mandate were similar in that they would have required that FP services be added as a covered benefit for designated health insurance plans. These first three versions of the legislation either died in committee or were vetoed by the governor amid concerns that they exceeded the essential health benefit ceiling set by the Affordable Care Act.^6^ SB 600, on the other hand, defined “standard FP services” as “basic healthcare services” which are required to be covered in all relevant health plans per pre-existing state law.^26^ It also clarified that “standard FP services” are defined as “procedures consistent with the established medical practices and professional guidelines published by the American Society of Clinical Oncology (ASCO) or the American Society for Reproductive Medicine (ASRM).” Furthermore, the language stated that SB 600 would not apply to Medicaid enrollees.

We assessed the innovation’s dynamism (i.e., potential for permanence). SB 600 defined FP services as “basic healthcare services” required to be covered under current law, thereby improving the potential for permanence of the FP benefit mandate. Conversely, when a benefit mandate is added as a stand-alone statute (as opposed to part of current law), it is easier for policy makers to introduce future legislation removing FP services from the list of state mandates or to include a sunset date for the policy. The policy developers wrote SB 600 specifically in this way to try to prevent non-compliance from impacted insurers. In addition, the reference to external guidelines from the ASCO and ASRM to define “standard fertility preservation services” allows for the policy to evolve as additional treatments become standard of care.

#### FP Policy Outcomes

Policy developers delineated the service outcomes (access to FP services, reduce denials of coverage for FP services and any related delays in providing cancer treatment) and long-term health outcomes (quality of life based on family building ability) but did not specify implementation outcomes.^27^ As researchers, we identified implementation outcomes and several additional service outcomes of SB 600 from the perspectives of stakeholders at each level (Table 1).

### 3. Identify and define the (non-linear) phases of policy D&I.

We identified key implementation processes across levels in nearly all EPIS phases (sustainment activities were rarely reported; Table 2). At the outer context regulator level, key processes included gathering stakeholder feedback in drafting regulator guidance, implementation via issuing the guidance and conducting independent medical reviews from consumers who were denied FP benefits, and assessment of compliance with regulations during sustainment. In a non-linear loop, stakeholder feedback during implementation and sustainment has driven preparation of additional regulator guidance on benefit specifics and populations covered. As of August 2023, these additional guidelines have not been open to public feedback or publicly issued.

At the inner context insurer level, implementation activities were documented across all four phases of EPIS. During the exploration phase, insurers reported monitoring potential legislation; gathering legal, medical, and actuarial expertise within the organization to shape insurer-level policies that would comply with the mandate; assessing compliance of existing contracts with purchasers/members, providers, and facilities; and evaluating capacity to administer the benefits. During the preparation phase, insurers reported 1) designing and selling FP benefits to purchasers; 2) ensuring adequate providers and facilities to deliver FP services; and 3) configuring staff and systems to administer FP benefits. Implementation phase activities included educating stakeholders about new benefits, performing benefit verification and pre-authorization, and processing claims and appeals. Sustainment activities such as monitoring and evaluation of patient utilization of FP benefits were less often mentioned.

Clinic staff reported engaging in exploration activities mainly through their participation in professional societies, whom they relied on to scan the environment and inform them of potential future policy changes. Clinic-level preparation activities included: 1) contracting with insurers to deliver FP services; 2) determining patient payment processes; and 3) configuring financial processes for interacting with insurers and patients. Contracting is time- and resource-intensive for clinics and does not occur when adequate reimbursement for services cannot be negotiated or patient volumes are expected to be low. The implementation processes that centered around accessing benefits were extremely complex. Thus, in non-linear loops after initial development, patient payment processes between the clinic and patient and financial processes between the clinic and insurer (benefit verification, prior authorization, claims, and appeals) were continually adapted in response to the many barriers encountered during attempts to utilize FP benefits. No sustainment-level activities were reported.

Temporally, the insurance regulator and insurers had nearly synchronous EPIS phases because regulator guidance was issued close to legislation passage (approximately 3 months), with the legislation going into effect immediately. In contrast, some clinics reacted to mandate passage later on as insurers reached out regarding establishing contracts, while most reacted even later as patients presented with FP service needs.

### 4. Describe the temporal roles that stakeholders play in policy D&I over time.

Actor roles across EPIS phases and domains are summarized in Table 2. Most actors have roles in more than one phase, and most of their actions span multiple levels. Across levels, exploration phase activities were primarily conducted by government relations personnel or external professional organizations that were relied on to monitor the environment and report on any significant proposed policy changes. This occurs in the inner context at the regulator level, at both the inner context and bridging context through professional societies at the insurer level, and through bridging activities only at the clinic level.

It was clear from interviews with stakeholders that individual characteristics of implementers in one level influenced implementation efforts across other levels. For example, clinic financial navigator expertise not only facilitated implementation at the clinic level but was also responsible for transfer of information to insurer benefit verification teams. In addition, expertise, relationships with other actors, and job tenure were noted as extremely important factors for implementation activities occurring across multiple levels (e.g., benefit determination, member education).

### 5. Consider policy-relevant outer and inner context adaptations.

**Figure 2:**
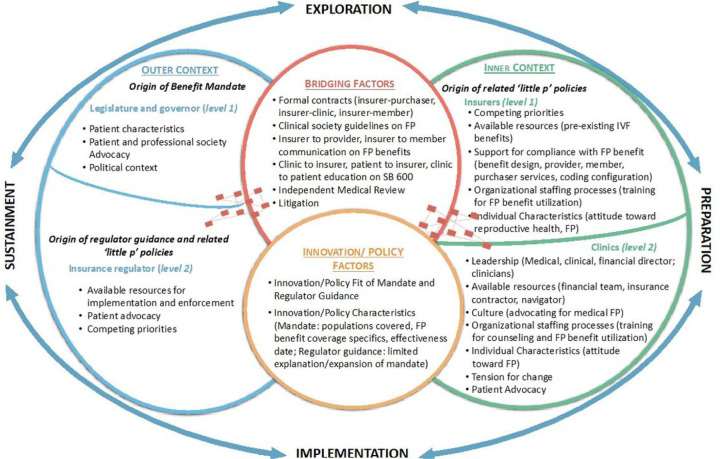
EPIS framework of contextual factors important to FP benefit mandate implementation. The policy innovation is the FP benefit mandate. Contextual factors within and between outer context (legislature, governor, and insurance regulator) and inner context (insurer and clinic) levels impact benefit mandate implementation.

Preparation activities primarily occurred in the inner context, while implementation activities took place in the inner context and through bridging factors between the inner and outer contexts. Data support that there are contextual factors within regulator-, insurer- and clinic levels that impact implementation (Fig. 2).

At the outer context regulator level, the most relevant construct that influenced implementation is competing priorities. Most of the time, no resources are allocated specifically for the implementation of state benefit mandates; therefore, the regulator may be under-resourced and unable to thoroughly engage in implementation activities. In California, implementation activities related to SB 600 have to compete with other pre-existing responsibilities, and the regulator may not have the ability to thoroughly evaluate, monitor, and enforce policies. Some states have started to explicitly allocate funds for implementation of benefit mandates to ensure that regulators have adequate resources to prioritize implementation activities.^14^

#### Insurer

The most relevant construct at the insurer level is also related to competing priorities. Insurers registered opposition for FP benefit mandate legislation but then needed to implement the policy after it became law. Therefore, it is unlikely that effective and eficient implementation is a top priority for the insurer. This may be even more pronounced for insurers that are for-profit and may have financial profits as a higher priority than ensuring patients have eficient and effective access to new treatments. In addition, as FP services are used by a small proportion of the population, promoting the new benefit will be of a lower priority than promotion of services used by a larger share of the population.

#### Clinics

Available resources and culture influenced FP financial practices with insurers and patients and ultimately FP benefit utilization. Nearly all participants discussed that the clinic’s financial team’s expertise is a key resource and the rate-limiting factor. Person power and experience are needed for contracting with insurers, benefit verification/billing coordinators, prior authorization, and billing/claims. These present significant financial costs to the clinic. When the amount of work to take insurance is too high, clinics do not contract, do not advise patients that there may be FP benefits, or do not provide enough support to utilize benefits.

Some clinics are motivated by a culture that “puts the patient first” or prioritizes patients who need medically indicated FP. These clinics actualize this culture with staffing for financial counseling and FP navigation, identification of an oncofertility team, and policies such as absorption of costs of FP consultations.

In smaller clinics, staff often have larger and overlapping roles. For example, a financial counselor may also be the head of finance for the clinic, meaning they pay clinic bills, order lab supplies, etc., or may also be the IVF coordinator, making them less effective in performing the role of financial counselor.

Experiences during implementation fueled tension for change by clinic financial teams, leading to modifying policies for patient payment and counseling and financial team training for FP patients. Some clinics changed patient payment policies. One clinic implemented a protocol to learn whether a patient’s insurance plan is subject to the mandate. If subject, even without benefit verification, the clinic required a small partial payment up front, relying on the ability to appeal after services are completed. Very few clinics had the ability to do so and instead implemented deferral of collections until all appeals are completed, while most clinics required full payment up front if there is no insurance benefit or insurer-clinic contract. The timing and frequency of financial communication may be important to helping patients make timely decisions on whether care is feasible while not overwhelming them. For clinics that accept insurance, benefit verification and financial counseling were often moved from after the initial medical visit to before the visit, due to the financial responsibility expected of the patients. Here, patients who do not have verified benefits will often drop out of care.

Larger financial counseling teams invested in training new team members, as turnover is frequent. Most experienced financial counselors discussed training on the job because responses within and between insurers on individual cases are so heterogeneous that training materials are dificult to generate. Only one clinic generated a spreadsheet that summarized benefit verification processes by common insurers. Instead, one-on-one mentoring communicated tips such as using cancer diagnosis rather than the FP code, because the latter is more likely to be treated as infertility, for which there is no insurance coverage.

### 6. Identify and describe bridging factors necessary for policy D&I success.

Key bridging factors were identified between all levels (Fig. 2). Bridging factors were identified as relationships between the outer and inner contexts, often reciprocal, that functioned to transfer knowledge between outer and inner context actors, contest the mandate’s scope across contexts, and ultimately promote policy transfer (clinic and insurance plan compliance with the mandate) and access to benefits. Two Big P’s (mandate, regulator guidance) give rise to many little p’s (e.g., independent medical review, bidirectional legal actions between the regulator and insurers, contracts) that served to bridge implementation and compliance with SB 600 across multiple levels and contexts within the health care system. Clinical society generated and endorsed clinical practice guidelines represented another bridging factor that influenced regulator (outer context) and insurer implementation (inner context).

Consistency of communicating benefit design to clinics and patients across different platforms – plan handbook, member services, provider services, web portals – was not met. Often, one or more of these bridging documents and resources lacked specificity regarding if and to what extent there are FP benefits. Often, two sources would provide discrepant information. This resulted in clinic financial staff undertaking time-consuming interrogation of all sources when clinic financial staff is a limited resource. One observed determinant of plan handbook accuracy is the timing of implementation. If mandates are signed late in a calendar year, plan handbooks for members may have already been written for the following year. The number of years since enactment may be a determinant of effective implementation.

Education on the benefit mandate was generated by insurers and some clinics, targeting insurers, clinics, and patients. Template letters to the insurer from clinics and patients included copies of the law and ASRM clinical guidelines that FP is standard of care. Provider bulletins and educational sessions were undertaken by insurers to both the clinic’s provider and administrative teams.

## Discussion

This study investigated FP benefit mandates as the evidence-based intervention to be implemented and followed the Big P policy through a complex, multi-level system. Guided by the policy-optimized EPIS framework and Bullock’s Policy Implementation Framework,^1,22,28^ this study contributes novel data to how the benefit mandate underwent implementation at and between California’s insurance regulator, insurers, and fertility clinics. This cataloging of key policies, processes, and actors serves to identify how big P and/or downstream little p policies are shaped or changed throughout implementation efforts and where policy-focused implementation strategies may be designed. As policy making is typically a long process that involves introduction of legislation multiple times prior to the final version passing, these data also provide opportunity for implementation considerations to be included in the policy making process to ensure effective implementation for access to FP services.

Consideration of the mandate’s function and form led to identification of downstream little p policies that were generated or adapted in response to SB 600 implementation. This finding encouraged cataloging little p functions (e.g., insurer-member, insurer-clinic communication on mandated benefits), with expanding understanding of their forms (e.g., member plan handbook, benefit verification processes) needed in the future to inform how the little p’s serve as determinants, implementation strategies, and/or mechanisms for mandate implementation. Determining the fundamental functions (i.e., purposes) of Big P and little p policies also informed asking about intended outcomes and cataloging a host of unintended implementation, service, and patient outcomes (e.g., bidirectional litigation, uncovered patient populations, patient medical financial hardship). These downstream policies and outcomes should be considered when measuring the effectiveness of FP mandates. In addition, the mandate did not allocate resources for implementation by regulators, including financial support for monitoring and enforcing compliance, which may have served as an additional barrier to implementation. Inadequate planning and collaboration with ‘downstream’ actors involved in policy implementation activities and compliance monitoring is a common catalyst of policy failure. Policy performance monitoring, managing, and capacity building are critical to successful policy implementation.^29^

An evaluation of the innovation’s structure found that while the mandate was dynamic enough to evolve over time, it was not specific enough to provide the guidance needed by stakeholders. The structure of the mandate was dynamic through definition of “basic healthcare services” by reference to ASCO and ASRM clinical practice guidelines. Despite this dynamism, due to a lack of specificity in the ASCO and ASRM guidelines regarding which treatments should explicitly be covered by health insurers, many insurers still lack the guidance needed to develop new benefit packages to fully implement the policy. Official documents that offer clearly worded guidance for interpreting complex health policies are critical to guiding multilevel actors in policy implementation efforts and achieving intended outcomes. For example, research assessing insurer compliance with the Mental Health Parity and Addiction Equity Act (MHPAEA) shows that individual state offices and insurers have differing legal interpretations of the law. Ongoing guidance with regularly updated Frequently Asked Question materials have been essential to ensuring nationwide compliance with MHPAEA rules across commercial and public payers.^30,31^ In our study, the absence of policy guidance resulted in a great deal of variation in the organizational “little p” policies developed to comply with the mandate. Future research can investigate the types of policy characteristics and policy-level dissemination strategies needed to promote clear communication about the intent of and compliance with a policy.

Delineating implementation processes by level and phase showed fairly synchronous regulator and insurer activities and a temporal, reactive lag in clinic activities. The synchrony appears to stem from the short timeframe between mandate passage to the time it was considered to be ‘in effect’ (immediately). This allotted limited time for the regulator to conduct rulemaking specific to the mandate and insurers to respond accordingly to both the mandate and regulator guidance. In contrast, a potential new regulator guidance on benefit specifics and populations covered (identified as necessary during the implementation and sustainment phases), has required preparation for more than two years and has yet to be implemented.

Temporally, clinic activities lagged because they were not subject to compliance with the mandate and were largely driven by patients’ FP service needs. The non-linear activity of preparing and implementing policies to access FP benefits was striking and in response to the heterogeneity of experiences with insurer provider and member services in accessing these benefits. Adaptations in the clinic’s inner context and bridging factors to insurers and patients followed but were heterogeneous among clinics, driven by available clinic resources and clinic culture/tension for change for this patient population. The non-linearity of implementation phases and associated adaptations were both characterized guided by policy-informed EPIS.

Little p policies generated downstream of the mandate served as bridging factors among all regulators, insurers, clinics, and patients. Among these, the essential bridging factor was contracts from insurers to purchasers, clinics, and members, but these contracts could be absent because of either the mandate characteristics (e.g., MediCal populations are not included), inner context (e.g., poor reimbursement offered), or poor fidelity to implementation (e.g., inconsistent benefit information to members and providers). Thematically, an additional purpose of multiple bridging factors was education: each level desired and designed policies to disseminate mandate and/or benefit information. Further data are needed on the effectiveness of these strategies. Finally, bridging factors including independent medical review and litigation served to test the scope of mandate.

Several limitations should be noted. During the study, ongoing legal action between the regulator and insurers prevented formal interviews of the regulator and some insurers, but we used public documents and other stakeholder data to assess these activities. We cataloged where little p policies were synthesized (or not) and heterogeneity among them, but how they impact fidelity to the Big P mandate and ultimately patients’ access to FP services has not been assessed and is the subject of ongoing work.

## Conclusions

Efforts to use policy, specifically state and federal health insurance benefit mandates, to reduce disparities in access to FP services has been ongoing for over a decade, culminating in 16 states with enacted mandates since 2017. Using implementation science frameworks adapted for health policy, this work documents the processes that occur after mandate passage, as well as defining actors and actions for those processes, in order to set the stage for evaluating determinants and important outcomes of effective implementation and designing future mandates and strategies to improve their implementation.

## Supplementary Material

Supplement 1

## Figures and Tables

**Table 1:** Implementation, service and patient outcomes from stakeholder perspectives

Source	Outcome	Perspective
***Implementation***		
Legislation (big P)	Lawsuits by insurers to state delay implementation of benefits	Regulator Insurers
Legislation (big P) and regulator guidance (big P)	Heterogeneity in benefit design in response to lack of FP services coverage specifics	Insurers
Insurer communication with members (little p)	Lack of or inconsistent FP benefit information through insurer member services, online member portals, evidence of coverage/plan handbook documents, and insurer communication with clinics	Clinics Patients
Insurer communication with clinics (little p)	Lack of or inconsistent FP benefit information through insurer provider services and portals, insurer communication with members	Clinics Patients
Heterogeneous insurer processes for benefit verification, prior authorization and claims (little p)	Time-consuming, parallel processes by clinics and patients for accessing FP benefits	Clinics Patients
Insurer system configuration of FP diagnostic and service codes and in network providers and facilities (little p)	Incomplete or errors in coding system lead to members and clinics misinformed that there is no benefit or not in network, clinics not getting reimbursed	Clinics Patients
Contracts between insurer and clinics (little p)	Lack of contracts or paired FP providers and facilities that are both in network for members give rise to need for letters of agreement for individual patients.	Clinics Patients
Payment requirements of patients (little p)	Clinics are unsure of insurance reimbursement and ask patients to pay cash costs up front	Clinics Patients
**Service**		
Legislation (big P)	Populations not covered (unisured, publicly insured, self-insured) renders policy ‘leaky’	Clinics Patients and advocates
Benefit design (little p)	Not all medically indicated FP services are covered, high out of pocket costs, and FP benefit not at parity with other benefits result in lack of access to and choice on services	Insurers Clinics Patients and advocates
Contracts between insurer and clinics (little p)	Few or no in network FP providers and facilities prevent access	Clinics Patients and advocates
Heterogeneous insurer processes for benefit verification and prior authorization (little p)	Without confirmed benefits, patients forgo consultation and treatments	Clinics Patients and advocates
Payment requirements of patients (little p)	Clinics are unsure of insurance reimbursement and ask patients to pay cash costs up front. Patients who cannot afford cash costs forgo services.	Clinics Patients
Dissemination of information on legislation and covered FP benefits	Providers may not offer and patients may not access FP services if they do not know that there are insurance benefits	Clinics Patients and advocates
**Patient and long-term health**		
Benefit design (little p)	High out of pocket costs result in patient distress, financial toxicity, and behaviors such as mortgaging homes to pay for FP services	Clinics Patients and advocates
Heterogeneous insurer processes for benefit verification, prior authorization and claims (little p)	Time-consuming and lack of resolution result in patient distress, financial toxicity	Clinics Patients

**Table 2:** Mandate implementation processes by level, EPIS phase and domain, and key actors

Process	Phase	Domain	Key actor(s)
***Regulator***			
Monitor proposed legislation	Exploration	Inner	Government relations
Meet with stakeholders and share draft guidance	Preparation	Bridging-stakeholders/public comment	Stakeholder relations
Issue guidance	Implementation	Inner	Deputy Director, Office of Plan Licensing
Conduct independent medical review	Implementation	Bridging – patients, insurer	IMR team; independent doctors
Review and revise regulator guidance	Sustainment	Bridging - insurer	Deputy Director, Office of Plan Licensing
Enforcement	Sustainment	Bridging – insurer	Office of Enforcement
**Insurer**			
Monitor proposed legislation	Exploration	Inner	Government relations
Compliance of existing benefits/plans	Exploration	Inner	Compliance dept.
Evaluate network capacity for services	Exploration	Inner	Provider relations
Evaluate costs of new FP benefits	Exploration	Inner	Health insurance actuary
Plan how to comply with SB 600 and regulator guidance	Preparation	Inner	Compliance dept.
Configure system to incorporate FP codes for benefit verification, pre-authorization and claims	Preparation	Inner	Member services, provider services, claims
Incorporate FP benefit into plan handbooks, member online portal, member service scripts	Preparation	Inner	Member services
Train member services, provider services, claims team on FP benefit	Preparation	Inner	Member services, provider services, claims
Contract with providers and facilities for FP services	Preparation	Bridging – clinic	Provider services
Sell/modify FP benefits to purchasers	Preparation	Bridging - purchaser	Sales and account management
Provider/clinic education on FP benefits	Implementation	Bridging – clinic	Provider services
Benefit verification	Implementation	Bridging - clinic	Utilization management
Administer benefit verification, prior authorization, claims processes	Implementation	Bridging - clinic	Utilization management
Answer member questions	Implementation	Bridging - patient	Member services
Generate letters of agreement	Implementation	Bridging – clinic	Provider services
Evaluate utilization	Sustainment	Inner	Quality Dept.
**Clinic**			
Learn about FP benefit mandate through clinical societies	Exploration	Bridging – professional clinical society	Clinician
Advocate for clinic adoption of financial and patient experience processes that enable benefit utilization	Exploration	Inner	Clinician, financial team
Negotiate contracts with insurers	Preparation	Bridging - insurer	Contracting specialist
Advocate for fertility benefit reimbursement rates at insurance contracting	Preparation	Bridging – insurer	Medical or clinic director
Determine patient payment options	Preparation	Inner	Medical director, clinic director, financial team
Allocate financial resources to staff financial navigation	Preparation	Inner	Medical or clinic director
Configure or modify processes for financial counseling of and collecting payments from FP patients • Defer payments if expect success in appeal • Convert visits to no charge because cannot wait for pre-authorization and appeals • Require patients to pay cash costs up front due to uncertainty of reimbursement	Preparation	Inner	Clinic director, physician, financial team
Train financial counselors • Processes for financial counseling of and collecting payments from FP patients • Insurer-specific processes	Preparation	Inner	Financial team
Generate tips, loopholes for financial team specific to insurers to disseminate among financial counselors	Preparation	Inner	Financial team
Generate tools for patients to interact with insurers • Lists of ICD, CPT, NPI, tax ID codes for patients to inquire with insurers • Benefit verification, appeal documents	Preparation	Inner	Financial team
Modify processes to conduct benefit verification be fore patient arrives, different from infertility patients	Preparation	Inner	Financial team
Benefit verification (online insurer portal, telephone call, via patients; primary and secondary insurer, fertility benefit carve out plans); assess if subject to SB600	Implementation	Bridging - insurer	Financial team
Submit prior authorization via online insurer portal, request expedited review, outreach to provider relations team for individual cases	Implementation	Bridging - insurer	Financial team
Submit and process claims to insurer	Implementation	Bridging - insurer	Financial team
Escalate benefit verification, pre-authorization, appeals and claims to insurer supervisors	Implementation	Bridging - insurer	Financial team
Prepare appeals to insurer and regulator for independent medical review	Implementation	Bridging – insurer Bridging – regulator, patient	Financial team patient navigator
Counsel patients on out-of-pocket cost estimates, appeal options, maximize benefits, philanthropic resources	Implementation	Bridging – patient	Financial team, patient navigator
Follow up with patients on insurer processes (claims, appeals)	Implementation	Bridging – patient	Financial team, patient navigator
Conduct parallel processes for benefit verification, pre-authorization, claims and appeals for medical and pharmacy benefits	Implementation	Bridging – insurer	Financial team
Negotiate one-off letters of agreements for patients with benefits but out of network	Implementation	Bridging – insurer	Contracting specialist
Follow up on why not all plans with an insurer are included in a clinic’s contract with the insurer	Sustainment	Bridging – insurer	Contracting specialist
