# Supplementary material for: Implementation of state health insurance benefit mandates for cancer-related fertility preservation: Following policy through a complex system
Source: Res Sq. 2023 Oct 9:rs.3.rs-3340894. Preprint. [Version 1] doi: 10.21203/rs.3.rs-3340894/v1 (PMC10602193; doi:10.21203/rs.3.rs-3340894/v1)
Supplement: Supplement 1 [file NIHPPRS3340894V1-supplement-1.pdf]

Supplementary Files

This is a list of supplementary files associated with this preprint. Click to download.

- Guidelinesupplement.docx
